# Graphene oxide exacerbates dextran sodium sulfate-induced colitis via ROS/AMPK/p53 signaling to mediate apoptosis

**DOI:** 10.1186/s12951-021-00832-5

**Published:** 2021-03-25

**Authors:** Siliang Liu, Angao Xu, Yanfei Gao, Yue Xie, Zhipeng Liu, Meiling Sun, Hua Mao, Xinying Wang

**Affiliations:** 1 Department of Gastroenterology, Zhujiang Hospital, Southern Medical University, Guangzhou, 510515 People’s Republic of China; 2 Huizhou Medicine Institute, Huizhou, 516003 People’s Republic of China

**Keywords:** Graphene oxide, Colitis, Intestinal epithelial cell, Apoptosis, Reactive oxygen species, AMP-activated protein kinase

## Abstract

**Background:**

Graphene oxide (GO), a novel carbon-based nanomaterial, has promising applications in biomedicine. However, it induces potential cytotoxic effects on the gastrointestinal (GI) tract cells, and these effects have been largely uncharacterized. The present study aimed to explore the toxic effects of GO on the intestinal tract especially under pre-existing inflammatory conditions, such as inflammatory bowel disease (IBD), and elucidate underlying mechanisms.

**Results:**

Our findings indicated that oral gavage of GO worsened acute colitis induced by 2.5% dextran sodium sulfate (DSS) in mice. In vitro, GO exacerbated DSS-induced inflammation and apoptosis in the FHC cell line, an ideal model of intestinal epithelial cells (IECs). Further, the potential mechanism underlying GO aggravated mice colitis and cell inflammation was explored. Our results revealed that GO treatment triggered apoptosis in FHC cells through the activation of reactive oxygen species (ROS)/AMP-activated protein kinase (AMPK)/p53 pathway, as evidenced by the upregulation of cytochrome c (Cytc), Bax, and cleaved caspase-3 (c-cas3) and the downregulation of Bcl-2. Interestingly, pretreatment with an antioxidant, N-acetyl-L-cysteine, and a specific inhibitor of AMPK activation, Compound C (Com.C), effectively inhibited GO-induced apoptosis in FHC cells.

**Conclusions:**

Our data demonstrate that GO-induced IECs apoptosis via ROS/AMPK/p53 pathway activation accounts for the exacerbation of colitis in vivo and aggravation of inflammation in vitro. These findings provide a new insight into the pathogenesis of IBD induced by environmental factors. Furthermore, our findings enhance our understanding of GO as a potential environmental toxin, which helps delineate the risk of exposure to patients with disturbed intestinal epithelial barrier/inflammatory disorders such as IBD.

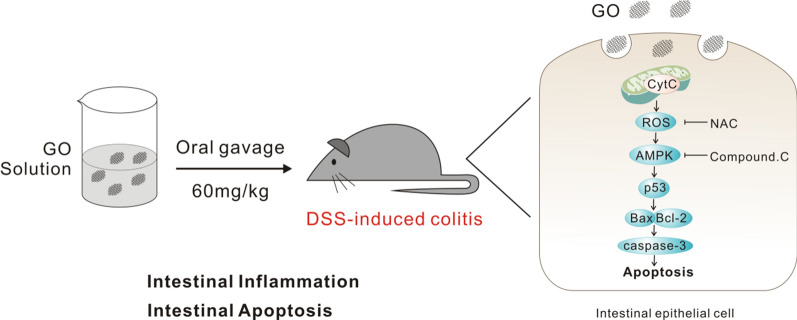

## Background

Inflammatory bowel disease (IBD) is a chronic, non-specific inflammatory condition of the gastrointestinal (GI) tract. The principal types of IBD are ulcerative colitis and Crohn’s disease [1]. In recent years, the incidence of IBD has gradually increased worldwide [2]. It is well documented that abnormal immunological reactions caused by genetic and environmental factors contribute to the development of IBD [3]. However, the exact underlying mechanisms remain unclear and require further investigation.

With the development of nanoparticle technology, the safety of nanoparticles has garnered substantial attention among researchers. Graphene oxide (GO), a promising derivative of graphene, possesses a large surface area and contains many surface functional groups compared with pristine graphene, which renders it as an attractive candidate for drug delivery, bone regeneration, antibiosis and even detection of pathogenic bacteria [4–8]. A previous study reported that the market for GO in 2020 could reach $618 million [9]. Along with an increase of GO in production and application, there has been an increase in concern over the unintentional or occupational exposure of GO and its subsequent impact on human health [10]. Until now, multiple studies have provided evidence that GO could be directly cell permeable or readily engulfed via endocytosis into tissues and cells, leading to the induction of adverse effects [11–13]. For instance, inflammation response in the lungs was observed in mice after GO exposure [14–16]. To the best of our knowledge, the toxic effects of GO on cells have been extensively studied and exposure to GO could cause a decrease in cell viability, alterations in the cell cycle, and apoptosis [17–19].

The intestinal tract is regarded as the primary site of interaction with nanomaterials, which renders the uptake of nanoparticles complicated because of the role of the intestinal barrier [20]. Although multiple studies have demonstrated the adverse impacts of GO on human health, only limited information on its effect on GI tract is available. In a systemic in vivo study conducted to detect the biodistribution of GO after intravenous injection and oral gavage, the results obtained suggested that the absorption of oral GO through the intestinal tract is ineffectual [15]. However, limited information still suggested that exposure to graphene family of nanomaterials alters intestinal barrier permeability by inducing apoptosis or changes in gut microbiota [21, 22]. Given the above findings, the exact toxic effects of GO on the intestinal tract and the underlying molecular mechanisms have not been systematically elucidated.

Intestinal epithelial cells (IECs) play a key role in maintaining the balance between the immune response and tissue homeostasis, especially as the apoptosis of IECs contributes to the chronic inflammation of the gut, such as during IBD [23]. Previous studies have provided evidence that GO-induced cytotoxicity promotes apoptosis through the activation of various signaling pathways, such as p38 mitogen-activated protein kinase signaling cascade and extracellular signal-regulated kinase signaling pathway [24, 25]. Adenosine monophosphate-activated protein kinase (AMPK), which is a conserved energy sensor, plays a crucial role in the antioxidant defense of cells and modulates cellular activities such as proliferation, cell cycle progression, and apoptosis [26]. Currently, the effects of GO on IECs apoptosis and the underlying mechanisms, such as the AMPK-related signaling pathway, remain unclear.

In the present study, we explored the potential toxicity of GO on the intestinal tract based on colitis induced by dextran sodium sulfate (DSS) and investigated the mechanism involved in vitro. Our study provides new insight into the pathogenesis of IBD, as it is related to environmental factors, and advances the current understanding of the risk of environmental exposure to GO.

## Results

### Characterization of GO

**Fig. 1:**
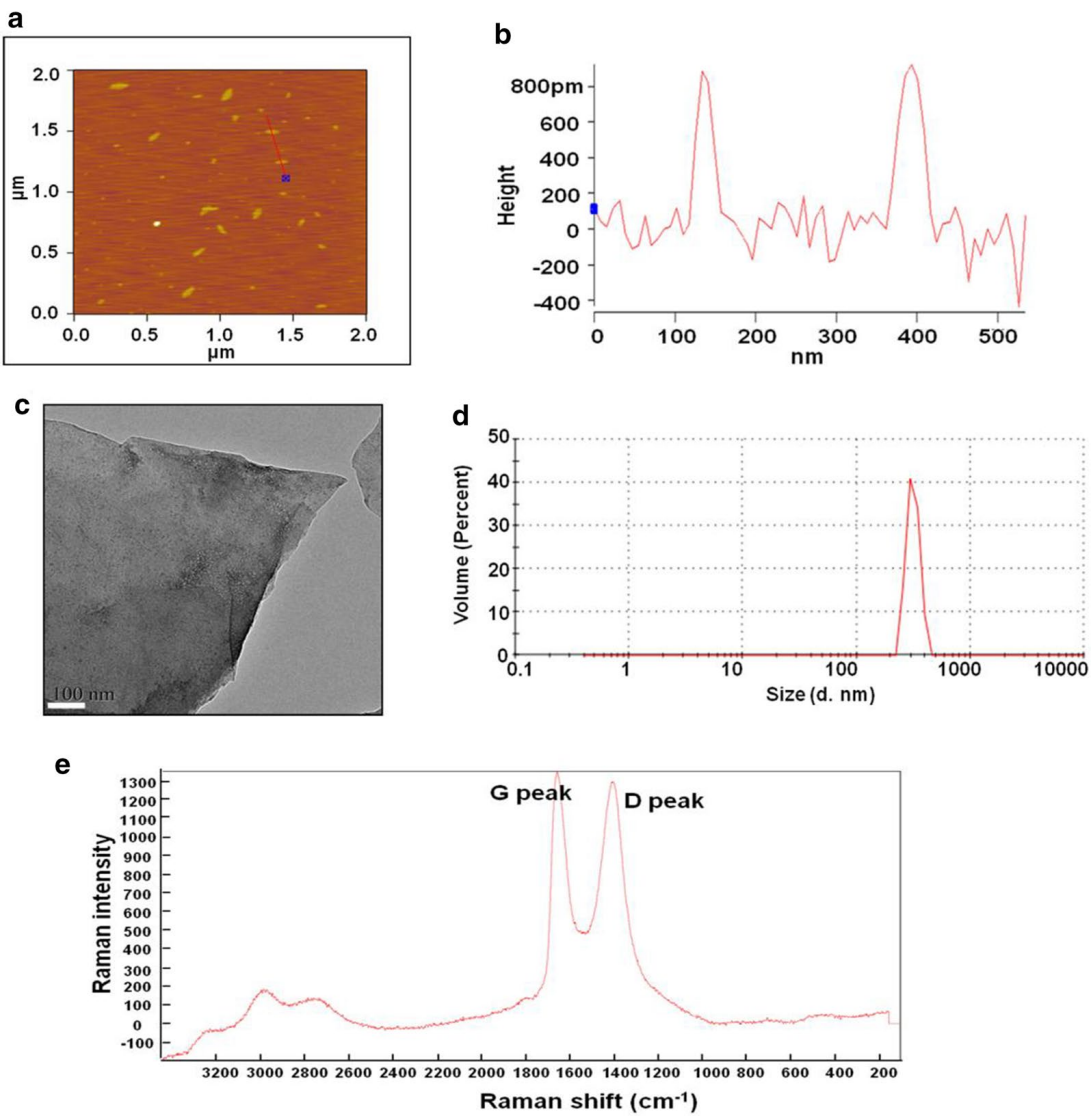
The characterization of GO. **a**, **b** Representative AFM images of GO. **c** Representative TEM image of GO. Scale bar: 100 nm. **d** The size distribution of GO in water using DLS. **e** Raman spectroscopy of GO nanosheets

To characterize GO used in this study, we used different microscopy techniques and observed our GO samples. Representative atomic force microscopy (AFM) images of GO are shown in Fig. 1a. Most GO were found to exist in a single layer or a few layers with a thickness of ~ 1.0 nm and a lateral dimension ranging from 200 to 300 nm (Fig. 1b), which is consistent with the basic characteristics of GO nanosheets. As depicted in Fig. 1c, GO also showed a monolayer structure with sharp edges via transmission electron microscopy (TEM). The size distribution of GO in water is shown in Fig. 1d. Based on the Raman spectra results, we significantly observed two distinctive D and G peaks at 1344 cm^−1^ and 1602 cm^−1^, respectively (Fig. 1e). Furthermore, we measured the average hydrodynamic particle sizes and zeta potentials of GO in different media using dynamic light scanning (DLS). As shown in Table 1, at 24 h, the average hydrodynamic particle size in water was 195.7 ± 0.8 nm, while in phosphate-buffered saline (PBS) and culture medium, it increased to 248.0 ± 1.4 nm and 333.5 ± 0.8 nm, respectively. Further, GO showed the most negative zeta potential in sterile water, followed by in PBS and culture medium, indicating GO was prone to aggregate in culture medium or PBS.

**Table 1:** Stability and dispersity of GO nanoparticles in different media

GO	In water		In PBS		In culture medium	
Time	Size (nm)	Zeta potential (mV)	Size (nm)	Zeta potential (mV)	Size (nm)	Zeta potential (mV)
12 h	159.9 ± 0.8	− 14.4 ± 2.6	95.4 ± 0.8	− 14.2	146.8 ± 0.2	− 7.1
24 h	195.7 ± 0.8	− 26.8	248.0 ± 1.4	− 12.5	333.5 ± 0.8	− 8.8
3 d	259.2 ± 0.7	− 16.5 ± 4.0	255.6 ± 2.1	− 17.0	321.4 ± 2.1	− 16.4 ± 0.8
5 d	294.8 ± 0.6	− 18.7	296.4 ± 0.9	− 14.6 ± 0.9	370.3 ± 2.4	− 9.4
7 d	311.8 ± 2.2	− 17.7	342.9 ± 1.2	− 17.1	1271 ± 3.9	− 3.4

*GO* graphene oxide, *PBS* phosphate-buffered saline

### Oral administration of GO nanoparticles aggravated DSS-induced colitis

**Fig. 2:**
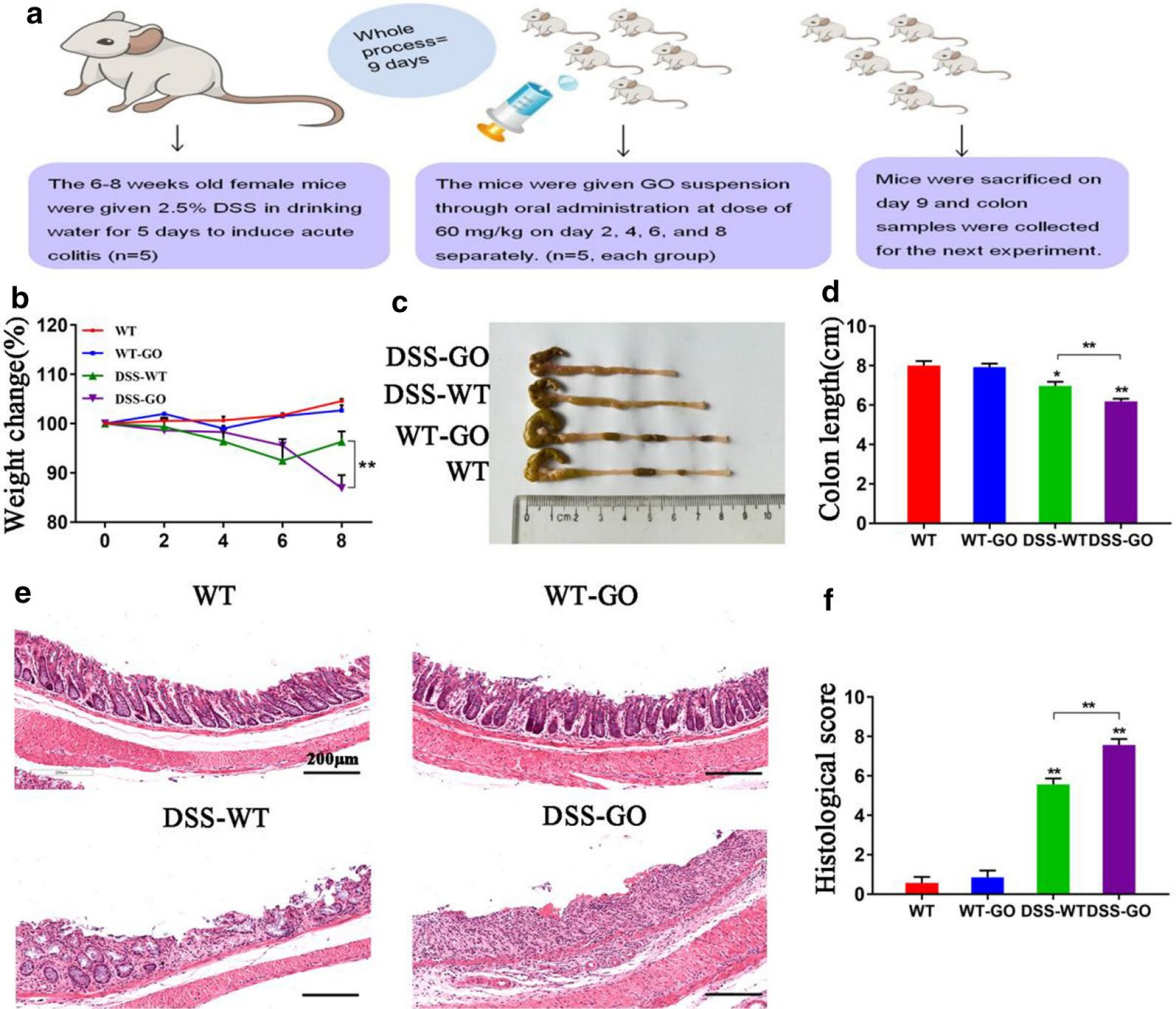
Administration of GO by oral gavage aggravated DSS-induced colitis in mice. **a** Schematic illustrations of experiments on the intestinal toxicity of mice after repeated oral exposure to GO. Mice were sacrificed on day 9 to collect colon samples. **b** Weight changes and **c**, **d** colon length were measured. Body weight was presented as the percentage of the initial weight (on day 0). **e** Representative H&E images of colon tissues. Scale bar: 200 μm. **f** The total histological score was calculated as the sum of the score for epithelial damage and the infiltration score. Data are presented as the means ± SEMs from three independent experiments in all analyses. *P < 0.05, **P < 0.01

To evaluate whether GO exposure affects colitis in vivo, we studied four experimental groups of mice. From days 4 to 6, the weight of mice in the DSS-wild-type (DSS-WT) and DSS-GO groups continuously decreased compared with that of mice in the WT and WT-GO groups. DSS-induced colitis in mice was observed to be a spontaneous limited disease, and the mice have gradually recovered from the weight loss after removing DSS-solution. However, after day 8, the DSS-GO group mice still showed a significant reduction in body weight compared with the DSS-WT group mice (Fig. 2b), and this was accompanied by an obvious shortening of the colon (Fig. 2c, d). In addition, hematoxylin and eosin (H&E)-stained sections of colonic tissues showed severe inflammatory cells infiltration (Fig. 2e), and the total histological score indicated severe disruption of the mucosal epithelium in the DSS-GO group, compared with DSS-WT group (Fig. 2f). Interestingly, mice that received GO in the absence of colitis showed no significant differences in weight, colon length, and histological scores from the corresponding parameters of mice in the WT group.

**Fig. 3:**
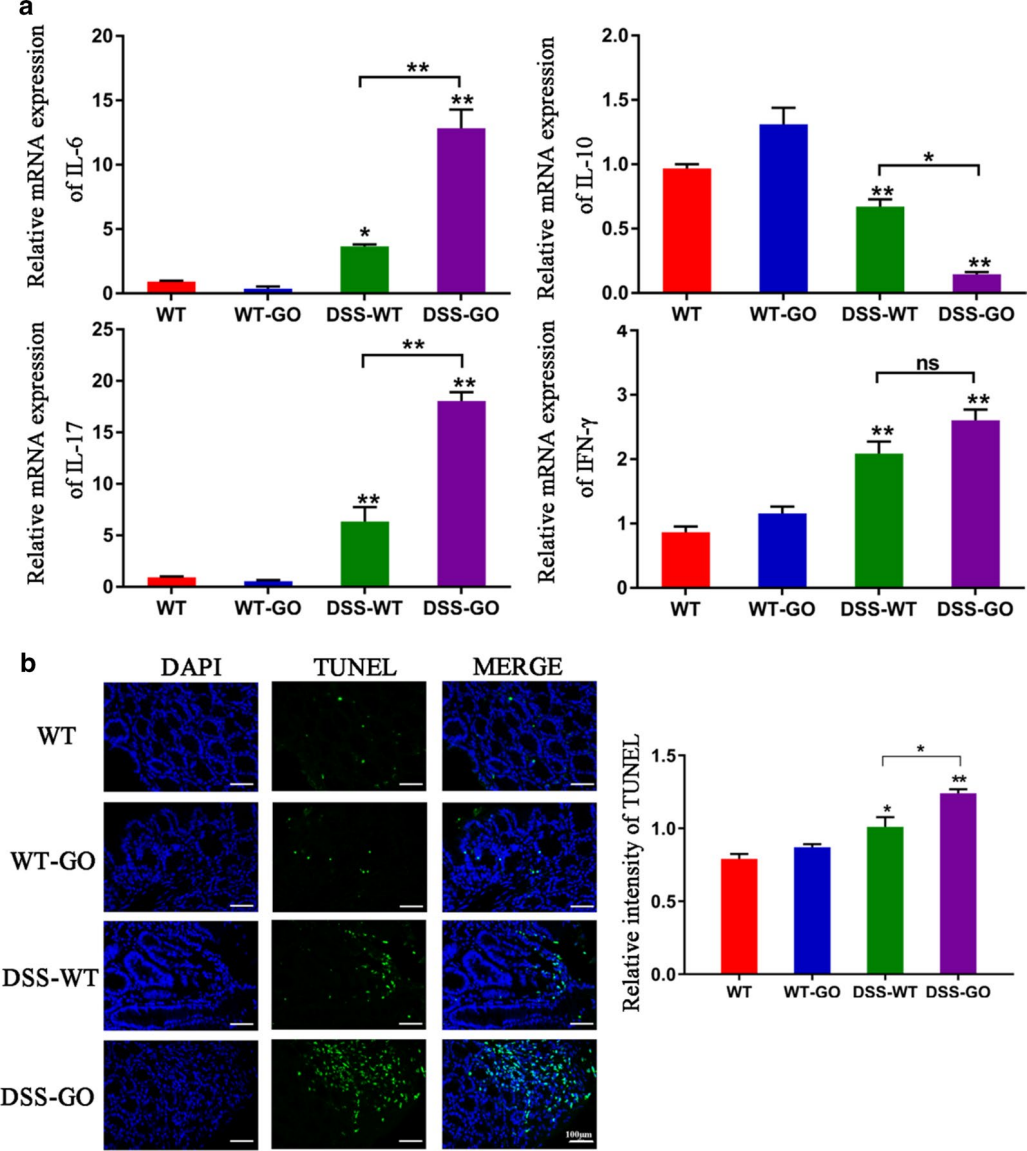
GO induced the production of pro-inflammatory cytokines and apoptosis in mice with DSS-induced colitis. **a** The relative mRNA expression of IL-6, IL-10, IL-17, and IFN-γ in mice was detected through qPCR. **b** Apoptosis in the intestinal tract was detected using TUNEL assay. Scale bar: 100 μm. *P < 0.05, **P < 0.01

To further evaluate the inflammatory response to GO in the intestinal tract, we detected the expression of several important inflammatory cytokines, which play an important role in colitis. Our results showed that GO treatment in the absence of colitis did not cause obvious changes in IL-6, IL-17, and IFN-γ expression (Fig. 3a). However, the expression of these pro-inflammatory cytokines increased in the DSS-WT and DSS-GO groups, while IL-10 expression decreased significantly (Fig. 3a). Besides, the DSS-GO group further promoted the release of pro-inflammatory cytokines than DSS-WT group.

For further research on the effects of GO on intestinal inflammation, we finally assessed intestinal apoptosis through terminal deoxynucleotidyl transferase-mediated nick end labeling (TUNEL) staining. Based on Fig. 3b, an obvious green fluorescence in the intestinal epithelium in DSS-GO group could be seen, compared with the DSS-WT group, indicating a higher rate of apoptosis in the former. This may have been the cause of exacerbating colitis but still required further investigation.

### The uptake of GO in IECs exacerbated cell inflammation induced by DSS

**Fig. 4:**
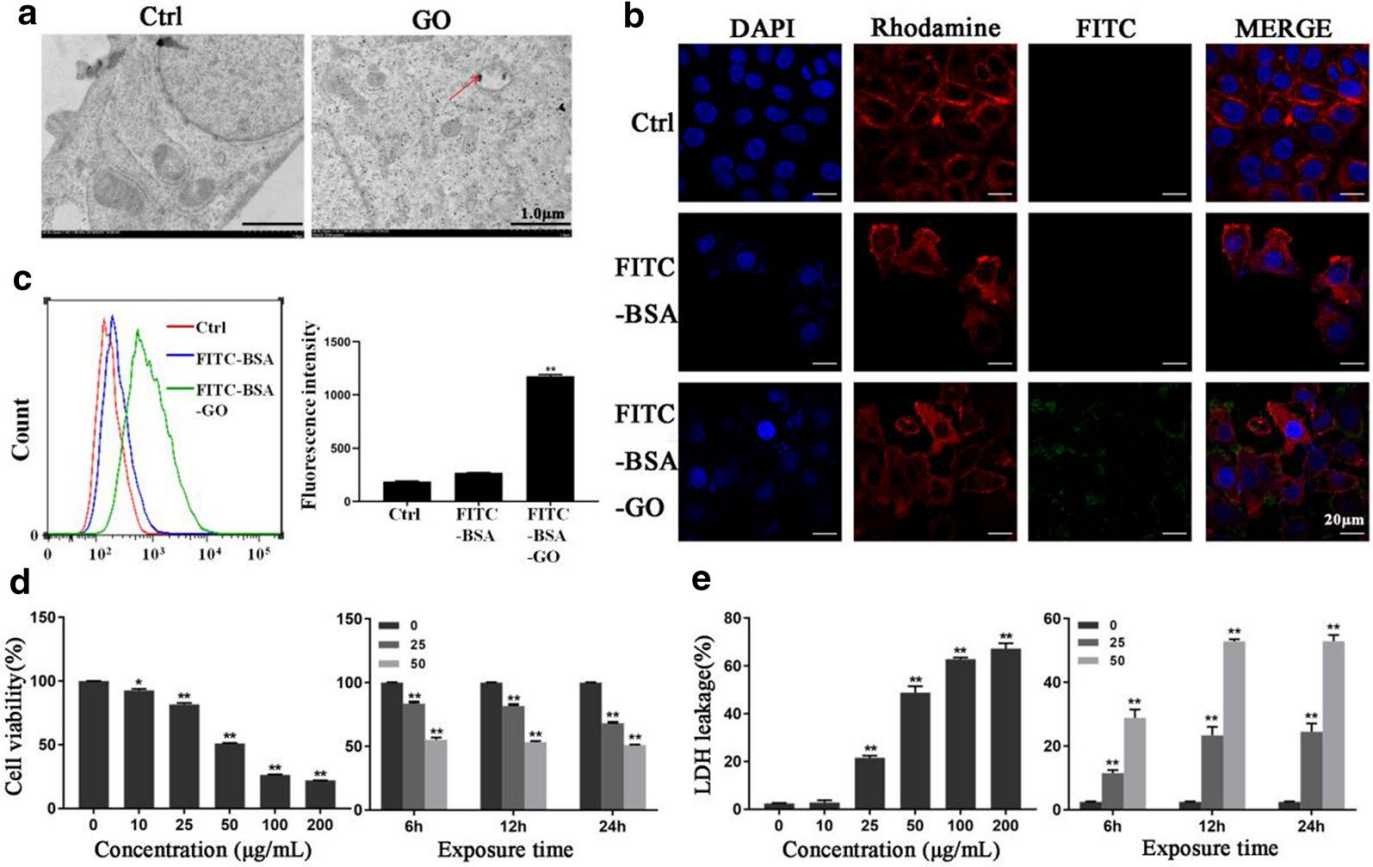
The uptake of GO in IECs induced dose- and time-dependent toxicity based on cell viability and LDH release. **a** The uptake of GO in FHC cells following 50 µg/mL GO treatment for 24 h. The red arrows indicate GO accumulation in the cytoplasm under TEM observation. Scale bar: 1 μm. **b**, **c** GO was labeled by FITC-BSA and exposed to FHC cells for additional 24 h. Untreated cells were served as control group, while single FITC-BSA exposed to FHC cells was included as negative control group. The green fluorescence of FITC-BSA labeled GO on the cytoplasm and cytomembrane of FHC cells using confocal microscopy. Scale bar: 20 μm. The relative fluorescence intensity was measured using flow cytometry. **d**, **e** Relative cell viability and LDH release in cultured FHC cells incubated with GO at different concentrations (0, 10, 25, 50, 100, or 200 µg/mL) for 24 h, or 25 and 50 µg/mL GO for 6, 12, and 24 h. The results were expressed as means ± SEMs  from three independent experiments. *P < 0.05, **P < 0.01

Given the aforementioned in vivo results, we next investigated the relationship between GO and IECs inflammation induced by DSS in vitro. First, we evaluated the biological activity of FHC cells, an ideal in vitro model of IECs, following GO treatment. As depicted in Fig. 4a, we found that GO was taken up into FHC cells and mainly distributed in the cytoplasm via TEM observation. Additionally, when compared with the control group and single fluorescein isothiocyanate (FITC)-bovine serum albumin (BSA) group, the FITC-BSA-labeled GO was detected on the cytoplasm of FHC cells after 24 h of incubation, as green-colored FITC-fluorescence signals, as shown in Fig. 4b. Meanwhile, a small amount of FITC-fluorescence was also detected in the cytomembrane. In addition, the results of flow cytometric analysis also showed that the fluorescence density of the FITC-BSA-GO group was significantly increased, compared to that in the FITC-BSA and control group (Fig. 4c), which further confirmed the absorption of GO by FHC cells. Subsequently, the potential toxic effects of GO on cell viability were detected. We found that GO significantly decreased FHC cell viability in a dose- and time-dependent manner (Fig. 4d). GO treatment at a concentration of 50 µg/mL for 24 h resulted in approximately 50% cell death compared with the cell death rate of the control group.

Next, the cell membrane integrity was evaluated using lactic dehydrogenase (LDH) release. It is seen from Fig. 4e that the LDH release from FHC cells significantly increased from almost 0 (control group) to more than 50% upon 50 µg/mL GO treatment. In addition, LDH release increased with a longer duration of GO exposure, which corresponds with the cell viability results. Based on the cell viability and LDH release results, we chose an appropriate concentration of GO (25 and 50 µg/mL) for subsequent experiments.

**Fig. 5:**
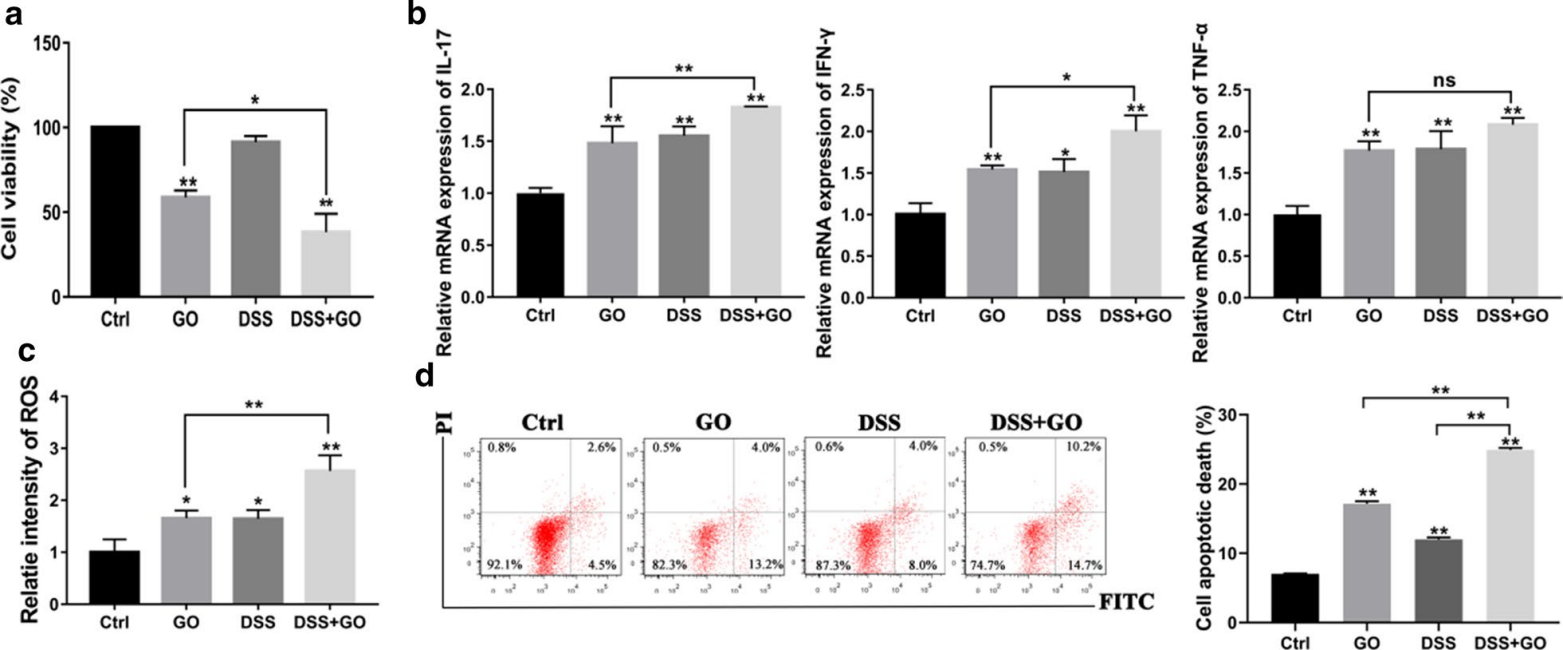
GO further exacerbated the cell inflammation induced by DSS in IECs. FHC cells were treated with 1% DSS for 0 or 4 h prior to culture in the presence or absence of GO. **a** CCK-8 was performed to assess the cell viability. **b** The relative mRNA expression of IL-17, IFN-γ and TNF-α using qPCR. **c** The intracellular production of ROS was tested using flow cytometry. **d** Flow cytometry was carried out to measure cell apoptosis. Apoptotic cell death was expressed as the sum of the percentage of early apoptotic cells and late apoptotic cells. The results were expressed as means ± SEMs from three independent experiments. *P < 0.05, **P < 0.01

For the purpose of verifying the potential effects of GO on intestinal inflammation in vitro, FHC cells were administered 1% DSS treatment prior to GO treatment to induce inflammation model in vitro. As shown in Fig. 5a, GO induced significant cell death (> 45%) compared to that in the control group after a 24-h exposure, whereas GO treatment in combination with DSS resulted in 58% cell death. Next, we investigated the inflammatory responses of FHC cells exposed to GO. As shown in Fig. 5b, GO and DSS both stimulated the release of IL-17, IFN-γ and TNF-α. Moreover, obvious inflammatory responses were observed in the DSS + GO group compared to that in the GO group, as evidenced by increased mRNA expression of IL-17 and IFN-γ. In addition, intracellular reactive oxygen species (ROS) production after GO treatment in the presence or absence of DSS was also tested. It was seen from the results of Fig. 5c that intracellular ROS production increased significantly after GO and DSS treatment. Moreover, GO further stimulated ROS generation with DSS treatment. Finally, we tested the effects of GO on apoptosis in FHC cells with or without DSS treatment. The results obtained showed that the percentage of apoptotic cells in FHC cells increased from 7.09% (ctrl) to 17.2% (GO group) and 12.02% (DSS group), which is shown in Fig. 5d. However, the percentage of apoptotic cells in the DSS + GO group reached 24.9%. These results are in line with in vivo results.

### GO induced mitochondrial dysfunction, ROS production and apoptosis in FHC cells

**Fig. 6:**
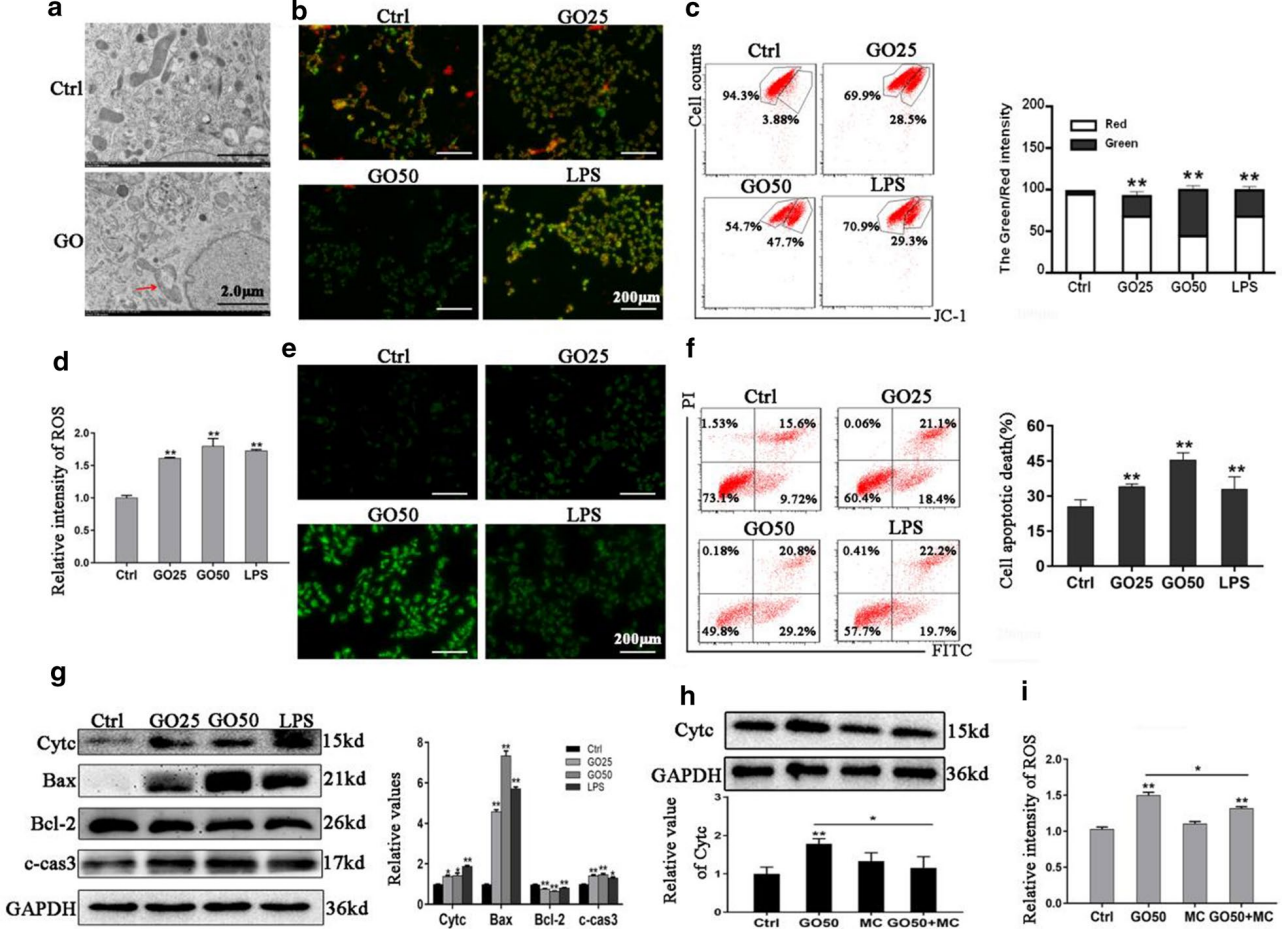
GO induced mitochondrial dysfunction, ROS production and cell apoptosis in FHC cells. **a** TEM observation of mitochondrial structure in FHC cells following exposure to 50 µg/mL GO treatment. Scale bar: 2.0 μm. **b**, **c** The MMP was analyzed via fluorescence microscopy and flow cytometry after GO or LPS treatment as indicated. Scale bar: 200 μm. **d**, **e** Flow cytometry and fluorescence microscopy were performed to measure intracellular ROS production. Scale bar: 200 μm. **f** Apoptosis of FHC cells after GO treatment as indicated. Apoptotic cell death was expressed as the sum of the percentage of early apoptotic cells and late apoptotic cells. **g** The levels of apoptosis-related proteins Cytc, Bax, Bcl-2, and c-cas3 using western blot. **h** Western blot of Cytc expression after incubation with GO in the presence or absence of MC. **i** Intracellular ROS production after GO treatment in the absence or presence of MC, as measured using flow cytometry. *P < 0.05, **P < 0.01

To explore the potential mechanism underlying the aggravation of colitis and cell inflammation mediated by GO, we further investigated the toxic responses to GO in FHC cells, focusing on cell apoptosis and the expression of related proteins. Firstly, we explored the mitochondrial structure and function following GO exposure. Apparent mitochondrial swelling and rupture were observed by TEM after GO exposure (Fig. 6a). Next, we tested the mitochondrial membrane potential (MMP), an important manifestation of early apoptosis, to evaluate mitochondrial function after GO exposure. Following GO treatment, we observed a GO-induced decrease of MMP (Fig. 6b, c). Moreover, intracellular ROS generation was detected. As shown in Fig. 6d, e, GO enhanced the levels of intracellular ROS. Cell apoptosis was also evaluated through flow cytometry. As shown in Fig. 6f, the percentages of apoptotic cells were 25.32% in the control group, 39.5% among FHC cells exposed to 25 µg/mL GO, an approximately 50% among FHC cells exposed to 50 µg/mL GO.

To reveal the potential mechanism responsible for cell apoptosis upon GO treatment, we detected the apoptosis-related proteins, with a focus on cytochrome c (Cytc), Bax, Bcl-2 and cleaved caspase-3 (c-cas3). As shown in Fig. 6g using western blot, GO treatment resulted in increased expression of Cytc, Bax, c-cas3 and decreased expression of Bcl-2, consistent with the induction of apoptosis.

Taken together, our results demonstrated that GO induced apoptosis, as evidenced by mitochondrial damage, Cytc release and ROS overproduction. To investigate the association between Cytc and ROS, we used minocycline (MC, an inhibitor of Cytc) before GO treatment and found that expressions of Cytc and intracellular ROS production were all reduced (Fig. 6h, i), which implied that GO-induced ROS generation resulted from mitochondrial dysfunction.

### GO-induced apoptosis was regulated via ROS generation through the AMPK/p53 pathway in FHC cells

**Fig. 7:**
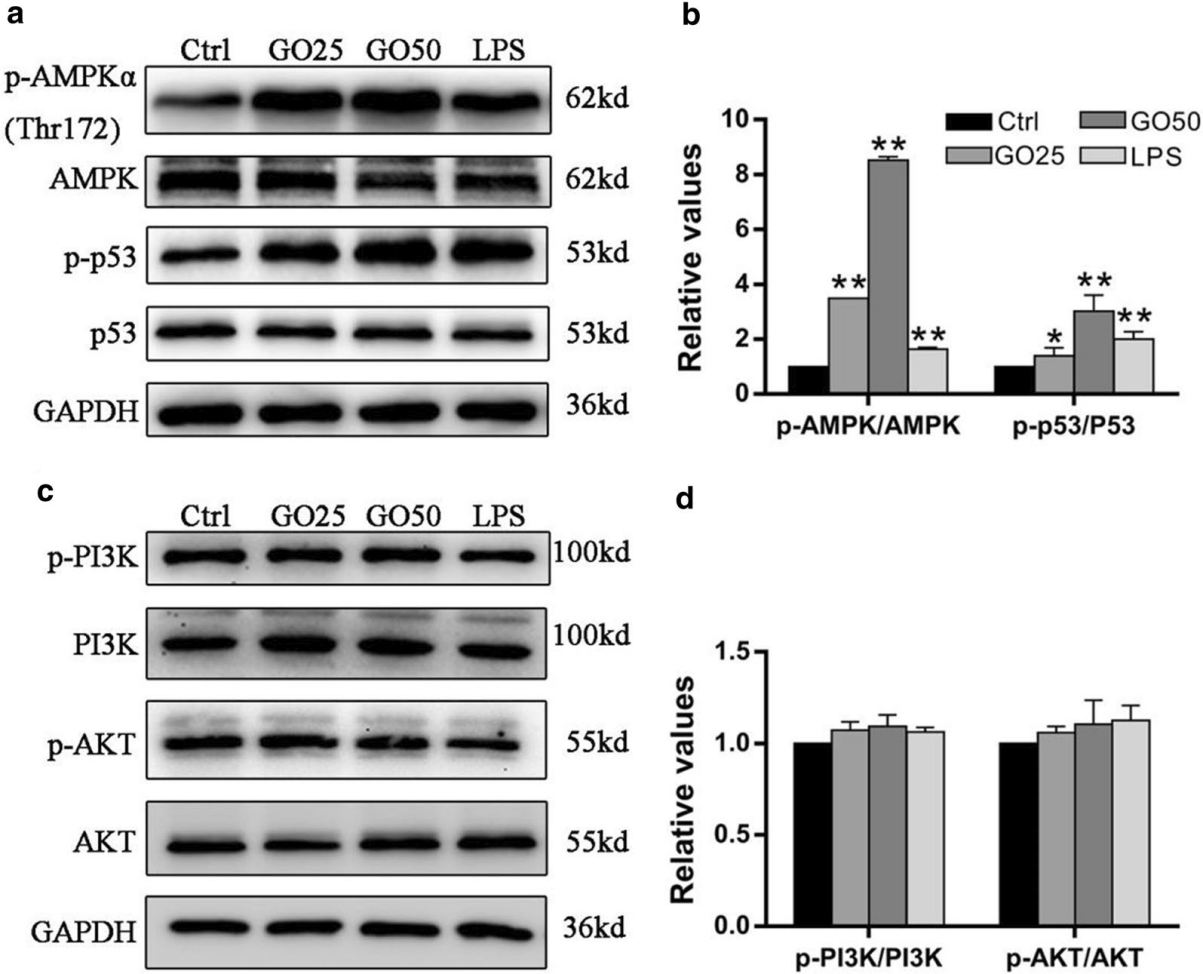
GO activated the AMPK/p53 signaling pathway in FHC cells. FHC cells were treated with 0, 25, or 50 µg/mL GO for 24 h, and LPS treated cells served as the positive control group. **a**–**d** Cell lysates were subjected to western blot to analyze the expression of p-AMPKα (Thr172), AMPK, p-p53, p53, p-PI3K, PI3K, p-AKT, and AKT. Changes in the levels of the phosphorylated proteins were quantified after normalization against the corresponding pan proteins. *P < 0.05, **P < 0.01

Given that the AMPK pathway plays an important role in cellular apoptosis, we investigated the effect of GO treatment on the expression of proteins involved in the AMPK pathway, including AMPK, PI3K, AKT, and p53. The levels of phosphorylated AMPK and p53 were significantly increased after GO treatment for 24 h (Fig. 7a, b), whereas the levels of phosphorylated PI3K and AKT were not (Fig. 7c, d).

**Fig. 8:**
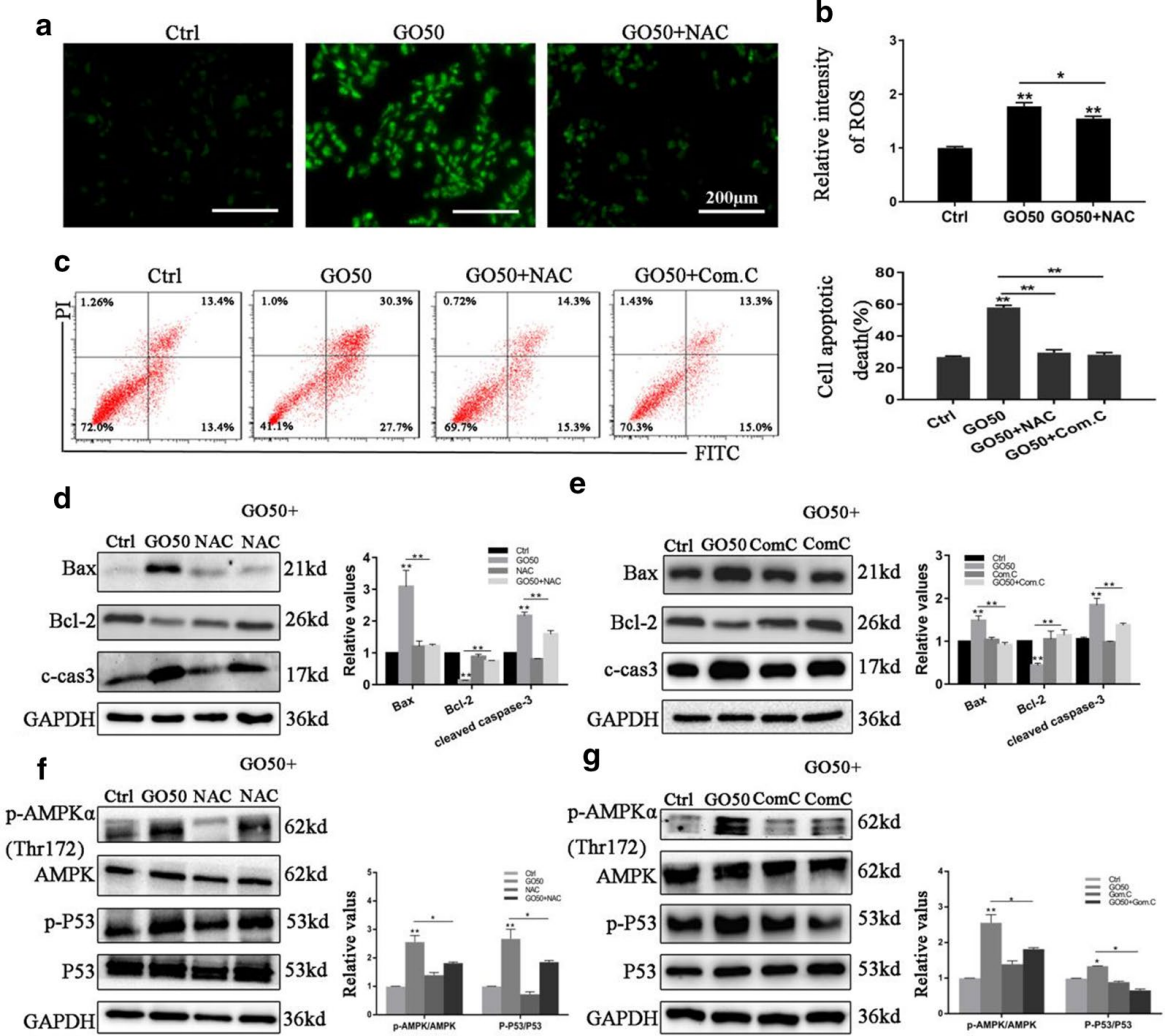
GO-induced apoptosis in FHC cells was regulated via the ROS/AMPK/p53 pathway. Cells were incubated with 50 µg/mL GO in the absence or presence of 400 µM NAC or 10 µM Com.C. **a**, **b** The intracellular ROS level was measured using DCFHDA detection through fluorescence microscopy and flow cytometry. Scale bar: 200 μm. **c** Flow cytometry was used to evaluate the apoptotic rate of FHC cells after exposure to GO. **d**, **e** The relative expression of Bax, Bcl-2, and c-caspase3 was measured using western blot. **f**, **g** The relative expression of AMPK, p-AMPK, p53 and p-p53 was measured following GO treatment in the absence or presence of NAC and Com.C. Changes in the levels of these proteins were quantified after normalization against GAPDH or AMPK and p53. *P < 0.05, **P < 0.01

To further examine the relationship among GO-induced apoptosis, ROS overproduction, and AMPK/p53 activation, we treated FHC cells with N-acetyl-L-cysteine (NAC), an ROS scavenger, and compound C (Com.C), a specific AMPK inhibitor, before incubation with GO. As shown in Fig. 8a, b, NAC significantly abrogated the GO-induced intracellular ROS accumulation. Additionally, flow cytometry showed significant reduction in GO-induced apoptosis after treatment of FHC cells with NAC and Com.C, from 58% to 29.6% and 28.3%, respectively (Fig. 8c). Moreover, pre-treating the FHC cells with NAC and Com.C resulted in the downregulated expression of c-cas3 and Bax and increased the protein expression of Bcl-2 (Fig. 8d, e). Finally, the western blot analysis showed that treatment with NAC and Com.C inhibited the phosophorylation of AMPKα (Thr172) and p53 in FHC cells (Fig. 8f, g). These results suggested that GO-induced apoptosis was regulated via the ROS/AMPK/p53 signaling pathway.

## Discussion

Herein, we aimed to find whether GO, a common environmental toxin, could negatively impact individuals with a defective intestinal barrier such as patients with IBD. We estimated the potential toxic effects of GO on a DSS-induced colitis mouse model and revealed the potential mechanisms in vitro. GO, a promising nanomaterial with a wide range of applications in biomedicine, has currently garnered tremendous research interest. Its extensive application means that the issue of potential GO toxicity worsens with the increasing environmental exposure. The intestinal tract is usually regarded as the primarily targeted organ by nanomaterials due to its direct exposure to the external environment. Nevertheless, the current understanding of the mechanism behind GO toxicity on the intestinal tract is poorly understood. Only a few in vivo studies were performed to address the hazard of GO following oral exposure [27–29]. Damage to the intestinal mucosal barrier is a defining characteristic of chronic intestinal inflammatory disorders such as IBD [30]. Based on the present findings, we proposed that there may be a close association between GO and chronic intestinal inflammation, which has not been studied/reported adequately. IBD has become a global health issue with its incidence continually increasing particularly in countries undergoing increasing westernization, thus underscoring the vital role of environmental factors [31].

In particular, the physiochemical properties of nanomaterials including the surface charge, lateral size, thickness, and aggregation status play an important role in the interactions with cells, organs, and tissues [32]. It is extremely critical for us to acquire detailed information on GO before evaluating its safety. For this purpose, we first characterized the GO used in this study. The results demonstrated that the commercial GO purchased from Sigma had a single to a few layers with sizes ranging from 200 to 300 nm. However, the hydrodynamic particle sizes of GO in PBS and culture medium gradually increased over time than those in water. This indicated that GO was prone to aggregation owing to the highly salted environment and the presence of protein components, which is consistent with the results of previous studies [33, 34].

Next, in vivo and in vitro experiments were carried out to assess the biosafety of GO. The in vivo results revealed that acute oral administration of GO increased the extent of colitis, accompanied by the release of pro-inflammatory cytokines (IL-6, IL-17, and IFN-γ) and apparent apoptosis in the intestinal mucosal epithelium. However, GO-treated mice in the absence of colitis did not show any inflammation in the intestinal tract compared with the mice in the control group, which is in line with a previous study [15]. It is noteworthy that other nanoparticles, including titanium dioxide and silica nanoparticle, have also been shown to increase intestinal inflammatory response [27, 35, 36]. Therefore, we hypothesized that the absorption of nanoparticles is enhanced significantly in the state of inflammation with a defective intestinal barrier, resulting in a further inflammatory response. These results suggest that GO is harmful in the presence of pre-existing inflammatory disorders, such as IBD. Furthermore, in vitro experiments were conducted to explore the potential mechanism for the role of GO in colitis.

In the present study, we focused on the direct toxic effects of GO on IECs, which play an important role in the development of chronic inflammation including IBD. Currently, most in vitro studies have chosen to use a human colon adenocarcinoma cell (Caco-2) line as a model to explore the interaction of GO with IECs [37, 38]. However, evidence indicated that the uptake of GO is closely related to the differentiation status of Caco-2 [39]. Therefore, in our study we chose the FHC cell line, which is also commonly used as an in vitro model of IECs, to explore the potential mechanism responsible for exacerbating colitis. Our data showed that the uptake of GO resulted in dose- and time-related alterations of cell viability, which correlates with the results of other studies in different cell models [17, 40, 41]. Considering that GO was also detected on the cytoplasmembrane of FHC cells using confocal microscopy, we also explored the effect of GO on cell membrane integrity and found obvious LDH release from FHC cells following GO treatment. Further, we found that GO exacerbated cell inflammation induced by DSS in FHC cells, as evidenced by decreased cell viability, elevated expression of IL-17 and IFN-γ, increased ROS production, and obvious apoptosis compared to those with GO treatment, which are consistent with the results of in vivo experiments. Furthermore, we explored the mechanism responsible for the aggravating effects of GO on colitis and cell inflammation. It is commonly accepted that the inflammatory response, DNA damage, apoptosis, oxidative stress, necrosis, and autophagy are involved in the toxicological mechanisms of GO [25, 42, 43]. Generally, intracellular ROS generation, reflecting the oxidative stress status, induces lipid peroxidation, protein inactivation, mitochondrial dysfunction, eventually leading to apoptosis [44]. Based on our data, the destruction of mitochondrial structure, decreased MMP, and increased level of intracellular ROS were observed in GO-treated IECs. We also observed notable apoptosis in IECs following GO exposure.

To the best of our knowledge, several apoptotic signals, such as DNA damage and cell stress as well as nanomaterial exposure, contribute to the activation of the Bcl-2 family of proteins and promote permeabilization of the mitochondrial membrane, further resulting in the release of pro-apoptotic proteins, such as Cytc and Bax. As a result of the formation of the apoptosome, caspase-9 and caspase-3 are activated, which then triggers apoptosis [45–47]. In our study, the GO-induced apoptosis is followed by the loss of MMP and the increasing in intracellular ROS level, which indicated that the mitochondrial pathway may be primarily responsible for this effect. To investigate this, we performed western blot, which showed that GO exposure caused increased Cytc, Bax, and cleaved-caspase3 expression, but decreased Bcl-2 expression. It has been suggested that Cytc is an essential molecular component of the electron transport chain, which promotes ROS generation [48]. In the present study, the inhibition of Cytc significantly prevented the intracellular ROS production induced by GO, which implied that GO-induced intracellular ROS accumulation resulted from mitochondrial dysfunction. Furthermore, prior studies have demonstrated that various signaling pathways are activated by ROS and numerous related proteins, such as MAPK, JNK and AMPK, are involved in the apoptotic process [49–51]. Notably, AMPK plays an important role in the regulation of apoptosis through modulating its downstream signaling molecules including p53, JNK, and mTOR [52–55]. To characterize the AMPK/p53 pathway involved, we measured the levels of AMPK and p53, as well as their phosphorylated forms. We found that GO activated the AMPK/p53 pathway. Furthermore, we found that Com.C treatment led to a significant decrease in apoptosis, which confirmed the crucial role of the AMPK/p53 pathway in GO-induced apoptosis. Cytosolic p53 translocates to the mitochondrial surface and directly interacts with Bcl-2 family of proteins, further leading to the release of Bax and the activation of apoptosis [56]. We also observed an increased expression of Bcl-2 and reduced expression of Bax after pretreatment with Com.C. Moreover, the fact that NAC effectively inhibited the activation of AMPK suggested that ROS was an upstream molecule of AMPK activation, which is consistent with a previous study [57].

As mentioned above, GO exposure to DSS-treated mice resulted in severe intestinal inflammation. Exposure of IECs to GO induced the loss of MMP and the generation of intracellular ROS. Subsequently, ROS activated the AMPK/p53 pathway to trigger apoptosis. This could be the main mechanism underlying the exacerbation of colitis by GO. However, concerning the underlying mechanism responsible for GO exacerbation of colitis, our study only focused on ROS release and IECs apoptosis. There might be other mechanisms of apoptosis that could be involved, and these need further investigation.

## Conclusions

In summary, our findings demonstrate that the oral administration of GO exacerbates DSS-induced acute colitis via the activation of the ROS/AMPK/p53 signaling pathway to mediate apoptosis in IECs. Our study expands the understanding of GO toxicity in the GI tract and provides new insight into the biocompatibility of graphene materials, indicating that further research is needed. In addition, our findings suggest that individuals with a pre-existing intestinal inflammatory condition, such as IBD, must be cautious when unintentionally exposed to GO or its derivatives.

## Materials and methods

### GO characterization

Commercially produced GO powder, purchased from Sigma-Aldrich (USA), was dispersed in pure water to prepare a stock solution (1 mg/mL). Before characterization and subsequent experiments, the stock solution was sonicated for 2 h (40 kHz, power 99%) using an ultrasonic processor (Biosafer, China). For characterization, the prepared GO sample was separately placed on mica and copper plates with 200 mesh grids for assessment via AFM (Bruker, USA) and TEM (Hitachi, USA). The structure of GO was assessed using Raman spectroscopy (Renishaw, UK) with a 514-nm laser. To acquire detailed information on its physicochemical properties, GO was dissolved in pure water, PBS, and complete culture medium for 12 h, 24 h, 3 days, 5 days, and 7 days. Then, the average hydrodynamic particle size and zeta potential were analyzed using DLS (Malvern, UK). For DLS measurements, 1.2 mL of GO sample solution in the cuvette accepted the light from the laser and the process of each sample was conducted at least 12 runs. The photodiode detector (Malvern, UK) was used to acquire the DLS signals and then processed with Zetasizer nanoapplication software (Malvern, UK).

### Animal experimentation

Female C57BL/6 mice (6–8 weeks old and weighing 18–20 g), purchased from the Animal Research Center of Southern Medical University (Guangzhou, China), were housed in a specific pathogen-free facility. The mice were divided into four groups (n = 5 per group): WT mice without DSS or GO treatment (WT group); WT mice treated with GO (WT-GO group); WT mice treated with DSS to induce colitis (DSS-WT group); and mice with DSS-induced colitis exposed to GO (DSS-GO group). To generate the acute colitis model, female C57BL/6 mice were administered 2.5% DSS (MP, USA) orally with drinking water for 5 days and then received normal drinking water for 3 days. Mice were exposed to GO separately via oral gavage at a dose of 60 mg/kg/day on days 2, 4, 6, and 8. During the process, the mice were monitored daily to observe for weight change, diarrhea, and rectal bleeding. A schematic representation of the animal protocol is provided in Fig. 2a.

### H&E and TUNEL staining assay

Mice were sacrificed on day 9, and colon samples were collected and fixed in 4% paraformaldehyde, sectioned, and stained with H&E for examination using light microscopy. Histological scoring was conducted following a previously described system [58]. For the TUNEL assay, colon slices were stained with Reagent 1 (TdT) and Reagent 2 (dUTP) at a mass ratio of 2:29 following the instruction of the TUNEL kit for 1 h before nuclear staining with 4′,6-diamidino-2-phenylindole dihydrochloride (DAPI, Beyotime, China). After washing with PBS thrice, fluorescence microscopy (Nikon, Japan) was conducted to observe the apoptotic cells in the intestinal tract and collect images (UV excitation wavelength 330–380 nm, emission wavelength 420 nm; FITC green light excitation wavelength 465–495 nm, emission wavelength 515–555 nm).

### Cell culture

A human colon epithelium cell line, the FHC cell line, purchased from the American Type Culture Collection (ATCC, VA) was used as an ideal in vitro model of IECs because it could form a confluent layer, thus allowing us explore the cell membrane integrity [59]. FHC cell line was maintained in RPMI-1640 supplemented with 10% fetal bovine serum (FBS), 100 U/mL penicillin, and 100 mg/mL streptomycin (Gibco, USA). FHC cells were cultured in a humidified atmosphere at 37 °C with 5% CO_2_.

### Cell viability and membrane integrity assay

Cell counting kit (CCK)-8 (Dojingdo, Japan) and LDH assays were performed to evaluate the cell viability and cell membrane integrity, respectively. FHC cells were seeded in 96-well plates at a density of 5 × 10^3^ cells/per well and incubated overnight for adherence. GO was introduced into cells at various concentrations (10, 25, 50, 100, and 200 µg/mL) for 24 h or incubated with 25 and 50 µg/mL GO for 6, 12, and 24 h to investigate the cytotoxic effect of GO on FHC cells in concentration and time aspects, untreated cells served as the control group. For DSS-stimulated inflammation in vitro, FHCs were administered 1% DSS dissolved in RPMI-1640 for 0 h (ctrl group) or 4 h (DSS group) prior to co-incubation with GO (GO and DSS + GO group), which was reported previously [59]. For the CCK-8 assay, at the end of treatment, the culture medium was removed and the cells were washed with PBS three times. Precisely 10 µL of CCK-8 working buffer was added to each well and incubated for an additional 1 h at 37 °C. The optical density of each well at 450 nm was read using a microplate reader (Molecular Device, USA). For the LDH assay, after 24 h of co-incubation with GO as indicated above, the 96-well plate was centrifuged (400 g, 5 min) and 60 µL of supernatant from each well was separately transferred to another 96-well plate following the manufacturer’s instructions. The optical density of each well was read using a microplate reader at 490 nm.

### Detection of inflammatory cytokines

Colon samples were collected for the detection of inflammatory cytokines. In vitro, 500 µL FHC cells were seeded on 12-well plates at a density of 5 × 10^4^ cells/well and incubated overnight. Then, cells were pretreated with 1% DSS for 0 h or 4 h, followed by 50 µg/mL GO treatment for another 24 h. Total RNA from colon tissues and cells were extracted using Trizol reagent (Gibco, USA) and quantified using the NanoDrop spectrophotometer (Thermo Fisher, USA). Quantitative real-time PCR (qPCR) was conducted and analyzed using LightCycler 480 (Roche, Switzerland). The inflammatory cytokine primers used are listed in Table 2.

**Table 2:** The primers of inflammatory cytokines used in the study

Gene name	Organism	Primer sequence
GAPDH	Mus musculus	GGGTCCCAGCTTAGGTTCAT
		TACGGCCAAATCCGTTCACA
IL-6	Mus musculus	TTCACAAGTCGGAGGCTTA
		CAAGTGCATCATCGTTGTTC
IL-10	Mus musculus	GGAAGAGAAACCAGGGAGA
		CCACAGTTTTCAGGGATGA
IL-17	Mus musculus	TTCACTTTCAGGGTCGAGA
		GGGGTTTCTTAGGGGTCA
IFN-γ	Mus musculus	ACTGGCAAAAGGATGGTG
		GTTGCTGATGGCCTGATT
GAPDH	Homo sapiens	CCTTCCGTGTCCCCACT
		GCCTGCTTCACCACCTTC
IFN-γ	Homo sapiens	CCGCTACATCTGAATGACCTG
		TGGCTTTTCAGCTCTGCATC
TNF-α	Homo sapiens	CCTCTCTCTAATCAGCCCTCTG
		GAGGACCTGGGAGTAGATGAG
IL-17	Homo sapiens	GGATGTTCAGGTTGACCATCAC
		TCCCACGAAATCCAGGATGC

### TEM observations of GO uptake and mitochondrial structure

Exactly 1 × 10^6^ FHC cells were cultured in 6-well plates and exposed to GO as indicated. The cells were collected via centrifuging after 24 h of incubation and fixed with 3% glutaraldehyde, post-fixed in osmium tetroxide, dehydrated in ethanol, and then polymerized using epoxy resin. GO uptake as well as the intracellular mitochondrial structure were observed via high-resolution ht7700 TEM (Hitachi, Japan).

### Confocal microscopy and flow cytometry of GO uptake

According to a previously established method [60], the prepared GO suspension was mixed with FITC-BSA (FITC/BSA = 5:1, Bioss Inc., China) at a mass ratio of 1:1 and incubated overnight at 37 °C in the dark. The mixture was centrifuged at 12,000×*g* for 30 min at 4 °C and washed briefly with PBS. Then, the pellet was resuspended in the culture medium and added to FHC cultures, which were seeded on sterile coverslips inside culture dishes. Twenty-four hours later, the cells were washed with PBS and fixed with 4% paraformaldehyde, followed by 0.1% Triton X-100 permeabilization. Finally, prior to nuclear staining with DAPI, cells were incubated with rhodamine phalloidin (100 nM) for cytoskeleton staining. After washed thrice with PBS, cells were observed under an FV1000 confocal laser scanning microscope (Olympus, Japan). FV10-ASW 3.0 Viewer software was used to analyze the acquired images.

Flow cytometry (BD Biosciences, USA) was also conducted to detect the uptake of GO by FHC cells. 1 × 10^6^ FHC cells were cultured in 6-well plates and then were exposed to FITC-BSA or FITC-BSA-GO, untreated cells served as control group. After 24 h of treatment, cells were collected and washed by PBS for 2 times, and then these samples were analyzed by flow cytometry immediately.

### Cell apoptosis assay

FHC cells were cultured in 6-well plates (1 × 10^6^ cells per well). To assess the potential toxic responses to GO with respect to cell inflammation, FHC cells were treated with 1% DSS for 0 or 4 h to stimulate inflammation at the cellular level, followed by co-incubation with or without GO (50 µg/mL) for an additional 24 h. To test the toxic effects of GO on IECs, FHC cells were incubated in the presence or absence of either GO (0, 25, and 50 µg/mL) or 10 µg/mL lipopolysaccharide (LPS, Sigma-Aldrich, USA) for 24 h. To further explore the potential mechanism in vitro, FHC cells were treated with 400 µM NAC (MCE, USA), or 10 µM Com.C (MCE, USA) for 1 h before GO incubation (0 or 50 µg/mL). After incubation as indicated, cells were harvested and washed with PBS three times followed by centrifugation (3000 rpm, 5 min). The cell pellet was suspended in 400 µL of binding buffer to achieve a density of 1 × 10^6^ cells/mL. The sample solution was then incubated with 5 µL Annexin V-FITC (Beyotime, China) for 15 min in the dark followed by an additional incubation with 10 µL propidium iodide (PI, Beyotime, China) for 5 min. Apoptotic cells were then detected using flow cytometry.

### MMP measurement

Briefly, cells were seeded in a 12-well plate at a density of 5 × 10^4^ cells/well and treated with GO at concentrations of 0, 25, and 50 µg/mL, and the cells treated with 10 µg/mL LPS were included in the positive control group. After exposure to GO or LPS, cells were incubated with JC-1 buffer mixture solution (Beyotime, China) for 20 min at 37 °C according to the manufacturer’s instructions. Fluorescence microscopy and flow cytometry were used to measure the ratio of green (JC-1 monomer)/red (JC-1 aggregates) fluorescence. The increased ration reflected the decrease of MMP.

### ROS generation assay

Intracellular ROS production was measured using the DCFHDA assay kit (Beyotime, China). Specifically, to explore the effect of GO on ROS generation under conditions of DSS-induced inflammation in vitro, 1 mL of FHC cells (at a density of 5 × 10^4^ cells/mL) was cultured in 12-well plates, incubated with 1% DSS for 0 or 4 h, and then exposed to GO treatment at 50 µg/mL. To detail the potential mechanisms, 1 mL FHC cells at density of 5 × 10^4^ cells/mL seeded in 12-well plates were separately treated with LPS and GO (0, 25, and 50 µg/mL) or treated with 100 µM MC (Selleck, USA) and NAC before incubaction with GO (50 µg/mL). At the end of treatment, FHC cells were harvested by centrifugation and stained with DCFHDA for 30 min in the dark at 37 °C. The fluorescence intensity was analyzed using fluorescence microscopy and flow cytometry.

### Western blot analysis

A total of 30 µg of protein was separated using 10% SDS-PAGE and transferred to a PVDF membrane, which was then blocked with 5% w/v BSA. Membranes were probed with the indicated primary antibodies including rabbit polyclonal antibodies against Cytc, c-cas3, Bcl-2, phosphorylated (p)-AMPKα (Thr172), p-PI3K, PI3K, p-AKT, AKT, p-p53, and p53 (Cell Signaling Technology, USA) along with mouse monoclonal antibodies against Bax and AMPK (Proteintech, China), and GAPDH was used to normalize protein expression. Appropriate secondary antibodies conjugated to horseradish-peroxidase (HRP) were then added and incubated for 1 h. The antigen-antibody complex was detected using an enhanced chemiluminescence reagent (Millipore, USA). The gray intensity of the bands on the western blots was analyzed using the ImageJ software (NIH, Bethesda, USA).

### Statistical analysis

The experimental data were presented as the mean ± standard error of the mean (SEM). Differences among the data for the different groups were analyzed using one-way ANOVA. *P*-values less than 0.05 and 0.01 were considered significant, as indicated.

## Data Availability

The datasets used or analyzed during the current study are available from the corresponding author upon reasonable request.

## Abbreviations

AMPK: Adenosine monophosphate-activated protein kinase
AFM: Atomic force microscopy
BSA: Bovine serum albumin
C-cas3: Cleaved caspase-3
CCK: Cell counting kit
Com.C: Compound C
Cytc: Cytochrome c
DAPI: 4′,6-Diamidino-2-phenylindole dihydrochloride
DLS: Dynamic light scanning
DSS: Dextran sodium sulfate
FITC: Fluorescein isothiocyanate
GI: Gastrointestinal
GO: Graphene oxide
HE: Hematoxylin and eosin
IBD: Inflammatory bowel disease
IECs: Intestinal epithelial cells
LDH: Lactic dehydrogenase
LPS: Lipopolysaccharide
MC: Minocycline
MMP: Mitochondrial membrane potential
NAC: N-acetyl-L-cysteine
PBS: Phosphate-buffered saline
PI: Propidium iodide
qPCR: Quantitative real-time PCR
ROS: Reactive oxygen species
SEM: Standard error of the mean
TEM: Transmission electron microscopy
TUNEL: Terminal deoxynucleotidyl transferase-mediated nick end labeling
